# Incidence, Prevalence, and Health Care Outcomes in Idiopathic Intracranial Hypertension: A Population Study

**DOI:** 10.1212/WNL.0000000000011463

**Published:** 2021-02-23

**Authors:** Latif Miah, Huw Strafford, Beata Fonferko-Shadrach, Joe Hollinghurst, Inder M.S. Sawhney, Savvas Hadjikoutis, Mark I. Rees, Rob Powell, Arron Lacey, William O. Pickrell

**Affiliations:** From Swansea University Medical School (L.M., H.S., B.F.-S., J.H., I.M.S.S., M.I.R., R.P., A.L., W.O.P.), Swansea University; Neurology Department (I.M.S.S., S.H., R.P., W.O.P.), Morriston Hospital, Swansea Bay University Health Board; and Faculty of Medicine and Health (M.I.R.), University of Sydney, Australia.

## Abstract

**Objective:**

To characterize trends in incidence, prevalence, and health care outcomes in the idiopathic intracranial hypertension (IIH) population in Wales using routinely collected health care data.

**Methods:**

We used and validated primary and secondary care IIH diagnosis codes within the Secure Anonymised Information Linkage databank to ascertain IIH cases and controls in a retrospective cohort study between 2003 and 2017. We recorded body mass index (BMI), deprivation quintile, CSF diversion surgery, and unscheduled hospital admissions in case and control cohorts.

**Results:**

We analyzed 35 million patient-years of data. There were 1,765 cases of IIH in 2017 (85% female). The prevalence and incidence of IIH in 2017 was 76/100,000 and 7.8/100,000/y, a significant increase from 2003 (corresponding figures = 12/100,000 and 2.3/100,000/y) (*p* < 0.001). IIH prevalence is associated with increasing BMI and increasing deprivation. The odds ratio for developing IIH in the least deprived quintile compared to the most deprived quintile, adjusted for sex and BMI, was 0.65 (95% confidence interval 0.55 to 0.76). Nine percent of IIH cases had CSF shunts with less than 0.2% having bariatric surgery. Unscheduled hospital admissions were higher in the IIH cohort compared to controls (rate ratio 5.28, *p* < 0.001) and in individuals with IIH and CSF shunts compared to those without shunts (rate ratio 2.02, *p* < 0.01).

**Conclusions:**

IIH incidence and prevalence is increasing considerably, corresponding to population increases in BMI, and is associated with increased deprivation. This has important implications for health care professionals and policy makers given the comorbidities, complications, and increased health care utilization associated with IIH.

Idiopathic intracranial hypertension (IIH) is a condition of unknown etiology that is strongly associated with obesity. IIH predominantly affects women of childbearing age and causes chronic disabling headaches, visual disturbance, and, in a minority of patients, permanent visual loss. The definitive management is weight loss but a minority of patients require surgery in order to preserve vision.^1,2^ People with IIH potentially have high rates of health care utilization due to their comorbidities, multiple specialist consultations, diagnostic tests, CSF diversion procedures, and complications related to CSF diversion surgery.

Given the increasing global burden of obesity and the strong association of IIH with obesity, it would seem plausible to assume that the incidence of IIH, as well as associated health care utilization, is increasing.^3,4^ Although there is some evidence to support this,^1,4^ overall there is a paucity of data regarding the epidemiology, health care utilization, and outcomes of people with IIH. To our knowledge, there have not been any large population-level studies of IIH in Wales.

In this retrospective cohort study, we aimed to determine the temporal trends of IIH incidence and prevalence in Wales and health care utilization associated with IIH. We also investigated the effects of socioeconomic deprivation and obesity on IIH epidemiology.

## Methods

This was a retrospective cohort study using anonymized, routinely collected Welsh health care data within the Secure Anonymised Information Linkage databank (SAIL) at Swansea University, Wales, United Kingdom.^5–7^ SAIL contains anonymized linked datasets from a range of Welsh health sources including hospital admission and demographic data for the complete Welsh population (3.1 million, 2017 population estimate) and primary care records for approximately 80% of the Welsh population. SAIL uses an established and validated split-file approach for anonymized data linkage.^5,6^

In the United Kingdom, primary care consists primarily of general practitioners (GPs). Patients with IIH can first present to their GP, optometry, neurology, or emergency department before being given a diagnosis by ophthalmologists or neurologists in secondary care.

To validate the accuracy of IIH diagnosis codes in routinely collected data, we anonymously linked a list of successive definite and probable IIH cases in 2016 and 2017 from Morriston Hospital Swansea (the regional neurosciences center) with SAIL data. The IIH cases were reviewed by clinicians and classed as definite if they met all IIH criteria or probable if they met the criteria but a record of fundoscopy at the time of diagnosis could not be found (table e-1, zenodo.org/record/4064064).^8^ We reviewed all patients admitted to Morriston Hospital in 2017 with an IIH ICD-10 diagnosis code in their hospital admission record and checked whether they had also been given a diagnosis of IIH by a consultant neurologist.

To identify patients with IIH throughout Wales, we selected patients who had been given primary care or secondary care IIH diagnosis codes (table e-2, zenodo.org/record/4064064) within the study window (January 1, 2003, to December 31, 2017).

We excluded likely cases of secondary intracranial hypertension (CNS malignancies, venous sinus thrombosis, and malignant hypertension) (table e-3, zenodo.org/record/4064064). We also excluded patients with less than 1 year of primary care data prior to diagnosis, with insufficient demographic data, and who were diagnosed on their death date (figure e-1, zenodo.org/record/4064064). We recorded bariatric surgery procedures and CSF diversion and revision procedures within the IIH cohort (see tables e-4, e-5, and e-6 for codes, zenodo.org/record/4064064). We calculated incidence and prevalence for every year within the study window and also calculated incidence and prevalence for each of the 7 health boards within Wales.

We recorded deprivation using the Welsh Index of Multiple Deprivation (WIMD), 2011 version, in which Wales is divided into 1,897 lower layer super output areas (LSOAs) containing an average of 1,600 people.^9^ Weighted scores from 8 domains, representing different types of deprivation, are aggregated to form a WIMD score for each LSOA. The following are the 8 domains that provide the weighted scores: income, employment, health, education, access to services, housing, community safety, and physical environment. Each LSOA in Wales has been ranked from most deprived to least deprived according to its WIMD score and then grouped into quintiles, with quintile 1 being the most deprived and quintile 5 being the least deprived.

We created a control cohort for the case (IIH) cohort by 3:1 matching on sex, age (week of birth), and WIMD quintile at time of diagnosis of IIH. For any analysis of the control cohort involving diagnosis date, the control was given the diagnosis date of the matched patient with IIH.

To measure obesity within the IIH and control cohorts, we used primary care body mass index (BMI) data measured during routine GP consultations. BMI is defined as body mass in kilograms divided by height in meters squared. Within the IIH cohort, most had at least 1 recorded BMI. For those with more than 1 recorded BMI, we used the BMI nearest to the diagnosis date. We excluded likely erroneous BMI values of less than 10 kgm^−2^ and greater than 70 kgm^−2^.

We used BMI data recorded in primary care (GP) and self-reported BMI data from the National Survey for Wales (NSW) to measure temporal trends in obesity. The NSW is completed on behalf of the Welsh Government and over 11,000 randomly selected individuals annually report on a range of health- and lifestyle-related issues.^10^ Both sources of BMI data are open to ascertainment bias. People with low or high BMI are more likely to have their BMI recorded by their GP and questionnaire respondents are likely to underestimate their weight. We therefore also used a mean of both GP and NSW BMI data to estimate temporal trends in obesity (BMI >30 kg/m^2^) rates in the Welsh population.

We recorded the rate of unscheduled hospital admissions in the IIH and control cohort. Unscheduled hospital admissions were defined as emergency department attendances or emergency/unscheduled hospital admissions (Patient Episode Dataset for Wales dataset).

### Statistical Methods

We calculated IIH prevalence by dividing the number of people with a diagnosis of IIH by the total number of people, with sufficient demographic data, registered with a GP within SAIL on July 1 each year. We calculated annual IIH incidence rates by dividing the number of new IIH diagnosis by the number of patient-years at risk in that time period.

We used a logistic regression model to compare the relative contributions of BMI and deprivation on the risk of developing a new diagnosis of IIH. In this model, we included every person with a primary care BMI measurement within the study period. A diagnosis of IIH within the study period was the binary outcome variable. For controls with multiple BMI measurements, we used the last recorded BMI before the end of the study period. For individuals with IIH, we used the BMI and deprivation quintile closest to diagnosis. We excluded any person who died during the study window.

We used a paired *t* test to compare changes in BMI measurements for individuals, proportion tests to compare proportions, and rate ratio tests to compare rates of hospital admission. We used R version 3.5.3 for statistical analysis.

### Standard Protocol Approvals, Registrations, and Patient Consents

All studies using SAIL data need independent Information Governance Review Panel (IGRP) approval. This study obtained IGRP approval (ref 0695). This study used anonymized, routinely collected data and therefore written informed consent was not required. The Research Ethics Service has previously confirmed that SAIL projects using anonymized, routinely collected data do not require specific UK National Health Service research ethics committee approval.

### Data Availability

The detailed anonymized patient data used in this study are potentially re-identifiable and therefore not directly available for sharing but are available within the SAIL Databank at Swansea University. All proposals to use SAIL data are subject to review by an IGRP. Before any data can be accessed, approval must be given by the IGRP. The IGRP gives careful consideration to each project to ensure proper and appropriate use of SAIL data. When access has been approved, it is gained through a privacy-protecting safe haven and remote access system referred to as the SAIL Gateway. SAIL has established an application process to be followed by anyone who would like to access data via SAIL (saildatabank.com/application-process).

## Results

We analyzed a total of 35 million patient-years of data (a mean of 2.3 million patient-years of data per year) during the study period (2003–2017).

### Validating Diagnosis Codes

We identified 153 successive individuals with an IIH diagnosis admitted to our neuroscience center in 2016 and 2017 (see Methods). In order to validate the accuracy of IIH diagnosis codes used in the routinely collected data, we anonymously linked these 153 individuals with IIH diagnoses (138 definite and 15 probable) to health care data within SAIL. A total of 149 of these cases had linked primary and secondary care data; all 153 had linked secondary care data. We were able to ascertain 125 out of our 149 individuals with definite or probable IIH using GP codes alone (sensitivity 84%), 130/153 (sensitivity 85%) using hospital codes alone, and 140/153 using a combination of GP and hospital codes (sensitivity 92%). A total of 141 out of all 153 individuals having a hospital ICD-10 code for IIH admitted to a neuroscience center in 2017 had a consultant neurologist diagnosis of IIH (specificity 87%; table e-7, zenodo.org/record/4064064). We used a combination of GP and hospital codes to ascertain cases with IIH for the remainder of the study.

### IIH Prevalence, IIH Incidence, and Obesity Trends

**Figure 1:**
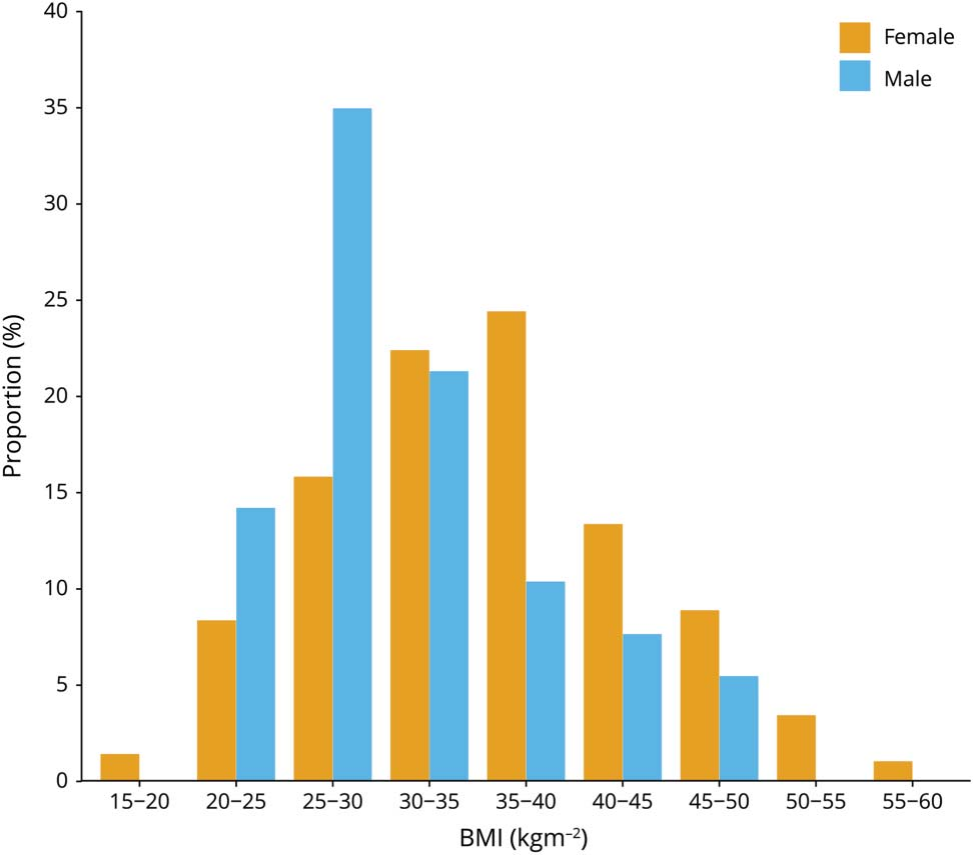
Body Mass Index (BMI) at Diagnosis for the Idiopathic Intracranial Hypertension (IIH) Cohort The distribution of BMI measurements at nearest IIH diagnosis for women (ochre) and men (blue). The y-axis shows the proportion of either all female or all male cases.

There were 2,275 people with primary or secondary care IIH diagnosis codes within the study window. After excluding potential secondary causes of intracranial hypertension and cases with no demographic data or no preceding primary care data, there was a prevalent IIH cohort of 1,765 in 2017 (figure e-1, zenodo.org/record/4064064; and table 1). A total of 86% of the cohort had a GP BMI recording at or after diagnosis of IIH (figure 1).

**Table 1:**
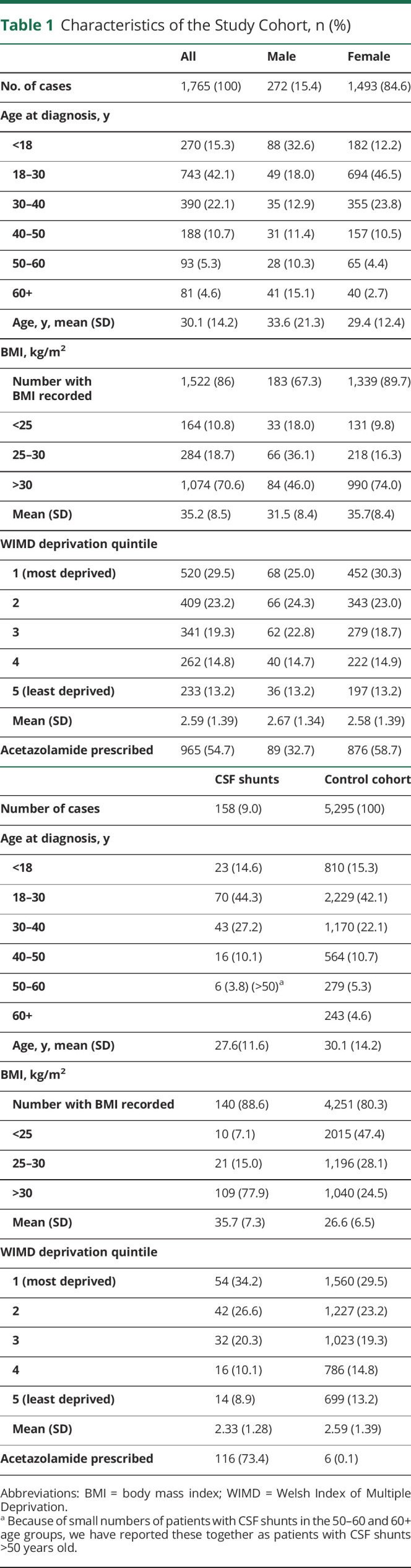
Characteristics of the Study Cohort, n (%)

**Figure 2:**
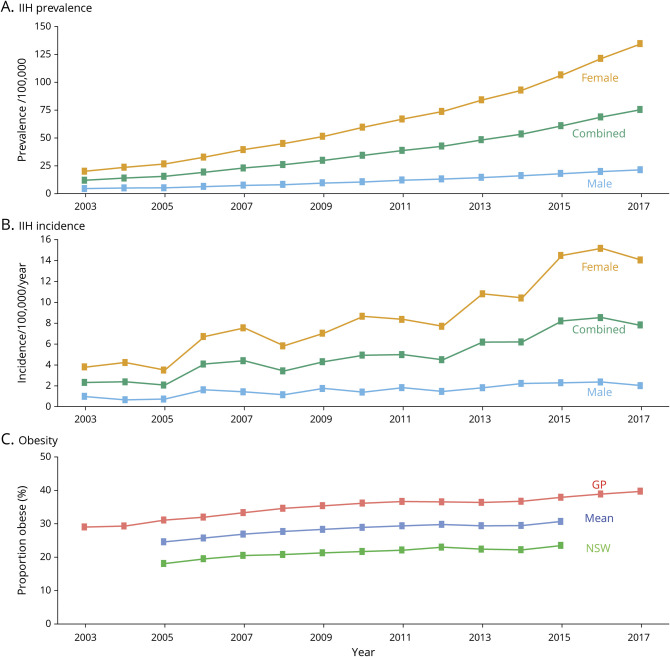
Prevalence and Incidence of Idiopathic Intracranial Hypertension (IIH) Compared With Obesity (2003–2017) Changes in (A) IIH prevalence and (B) IIH incidence with time during the study period. Ochre lines represent the female population, blue lines represent the male population, and green lines represent the combined population. (C) Changes in the proportion of the population who are obese (body mass index >30 kg/m^2^) from general practitioner (GP) BMI measurements (red line), National Survey for Wales (NSW) data (green line), and a mean average of the two (blue line) during the study period. The x-axis in all graphs represents time in years. Note NSW data were not available for 2003–2005 or 2015–2017.

**Figure 3:**
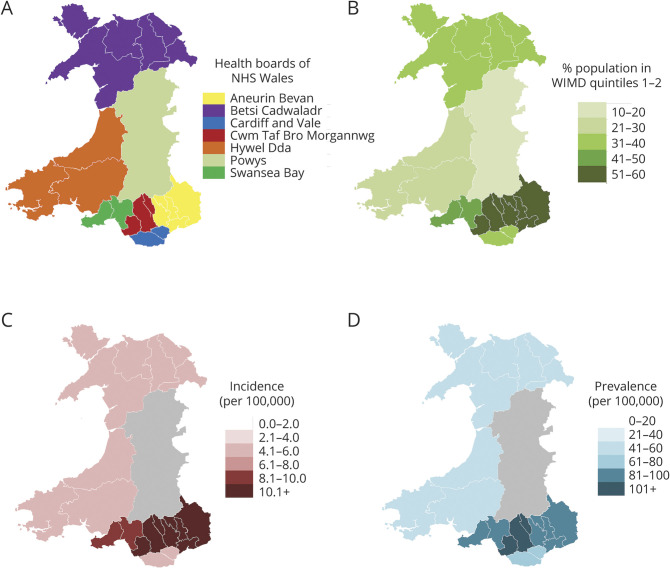
Choropleths Comparing Welsh Idiopathic Intracranial Hypertension (IIH) Incidence and Prevalence With Deprivation (A) Map of the current Welsh health boards. Choropleth maps show the distribution of (B) deprivation, measured as a percentage of the health board population in the 2 most deprived Welsh Index of Multiple Deprivation (WIMD) quintiles (1–2); (C) IIH incidence 2015–2017 by the health board; and (D) IIH prevalence in 2017 by the health board. Data for the Powys Health board could not be presented for incidence and prevalence due to restrictions in obtaining small numbers from Secure Anonymised Information Linkage. See also table e-8 (zenodo.org/record/4064064). NHS = National Health Service.

The prevalence of IIH increased greatly from 12/100,000 in 2003 to 76/100,000 in 2017 (*p* < 0.001) (figure 2A). The incidence of IIH increased greatly from 2.3/100,000/y in 2003 to 7.8/100,000/y in 2017 (*p* < 0.001) (figure 2B). The proportion of obese individuals (BMI >30 kg/m^2^) in Wales, as measured using primary care data, increased significantly, with 29% of the population being obese in 2003 compared to 40% in 2017 (*p* < 0.001). There were also significant increases in obesity as measured by questionnaire data (figure 2C). IIH incidence and prevalence by Welsh health boards is shown in figure 3 and table e-8 (zenodo.org/record/4064064).

### Association Among IIH, Deprivation, and BMI

**Figure 4:**
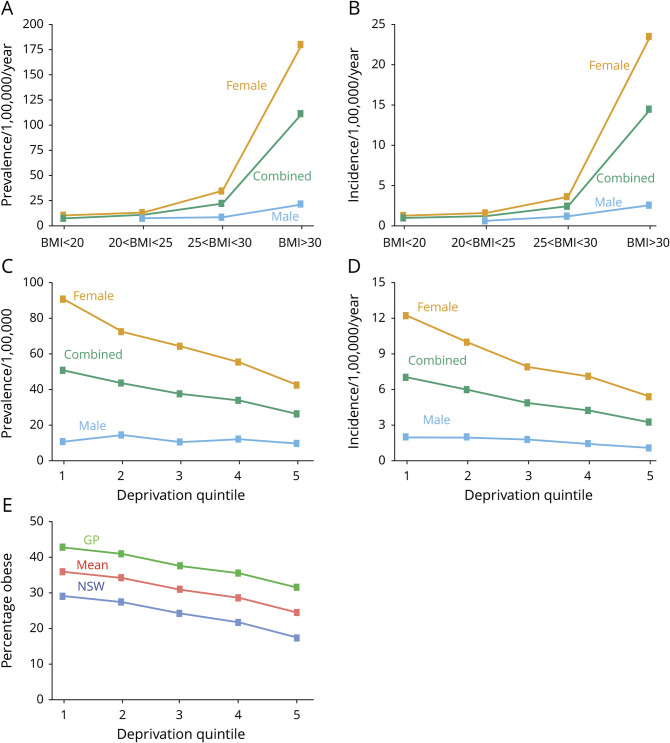
Relationship of Idiopathic Intracranial Hypertension (IIH) Prevalence and Incidence With Obesity and Deprivation Association between IIH prevalence (A) and incidence (B) with body mass index (BMI). BMI <20 kg/m^2^ is defined as underweight, 20 kg/m^2^ < BMI < 25 kg/m^2^ normal weight, 25 kg/m^2^ < BMI < 30 kg/m^2^ overweight, and BMI >30 kg/m^2^ as obese. (C, D) Relationship between IIH prevalence and incidence with deprivation as measured by the Welsh Index of Multiple Deprivation (1 = most deprived, 5 = least deprived). (E) Relationship between obesity and deprivation for 2015 (similar relationships exist for other study years; see figure e-2, zenodo.org/record/4064064). The green line is obtained from primary care BMI measurements, the blue line from Welsh Health Survey BMI data, and the red line is the mean of the 2 measurements. GP = general practitioner; NSW = National Survey for Wales.

Figure 4 illustrates the association between both IIH prevalence and incidence with obesity and the association between obesity and deprivation. Figure 4, A–D, use incidence and prevalence averaged over the whole study period and figure 4E uses obesity data for 2015 only. Similar patterns are seen for all years in the study period (figures e-2, e-3, and e-4; zenodo.org/record/4064064).

For both men and women, IIH prevalence and incidence are strongly associated with BMI. For obese women (BMI >30 kg/m^2^), the mean prevalence and incidence of IIH was 180/100,000 and 23.5/100,000/y. The corresponding figures for women with an ideal BMI (20 kg/m^2^ < BMI < 25 kg/m^2^) were 13.2/100,000 and 1.6/100,000/y, respectively. For obese men, the mean prevalence and incidence of IIH was 21.2/100,000 and 2.6/100,000/y. The corresponding figures for men with an ideal BMI were 7.6/100,000 and 1.6/100,000/y, respectively. In 2015, 35.9% of people in the most deprived quintile were obese, compared to 24.4% in the least deprived quintile.

Table 2 shows odds ratios for developing IIH based on sex, BMI, and deprivation score.

**Table 2:**
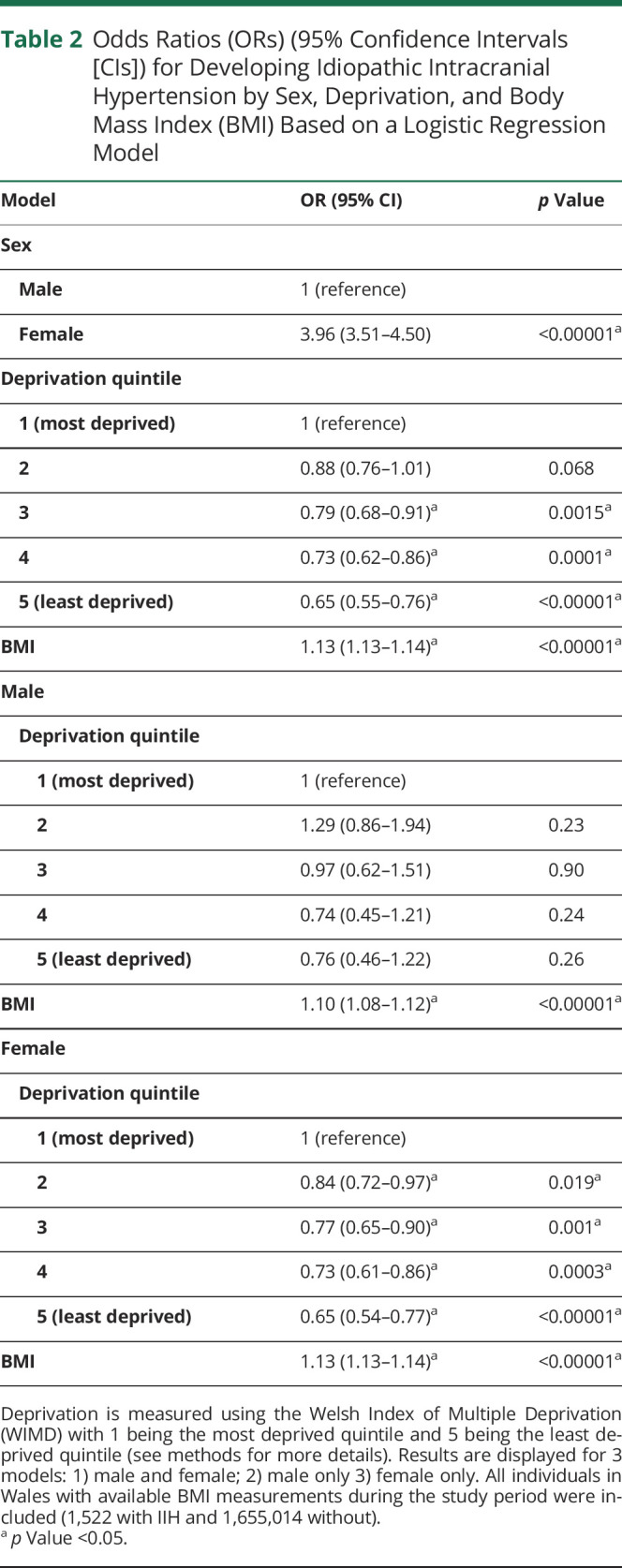
Odds Ratios (ORs) (95% Confidence Intervals [CIs]) for Developing Idiopathic Intracranial Hypertension by Sex, Deprivation, and Body Mass Index (BMI) Based on a Logistic Regression Model

### Outcome Data

Thirteen patients with IIH (0.78%) were recorded as being blind a mean of 858 days (28 months) after diagnosis. A total of 32 patients (1.9%) were recorded as having at least moderate visual impairment a mean of 804 days (26 months) after diagnosis. A total of 158 patients (9%) underwent CSF diversion procedures a mean of 491 days (16 months) after diagnosis (see table 1 for the characteristics of the shunt cohort and figure e-5 [zenodo.org/record/4064064] for a Kaplan-Meier plot). A total of 70 (44%) patients who had CSF diversion procedures went on to have at least 1 revision surgery.

A total of 691 patients with IIH had at least 2 BMI readings after their diagnosis (first BMI was within 1 year of diagnosis and the latest BMI reading was a mean of 5.1 years later). These patients' BMI increased by 0.48 kg/m^2^ (*p* = 0.02, 95% confidence interval [CI] 0.07 to 0.89). Fewer than 6 optic nerve fenestration procedures or gastric banding procedures were performed (note that due to SAIL anonymization guidance we cannot report groups of fewer than 6 individuals or events).

### Health Care Utilization

**Figure 5:**
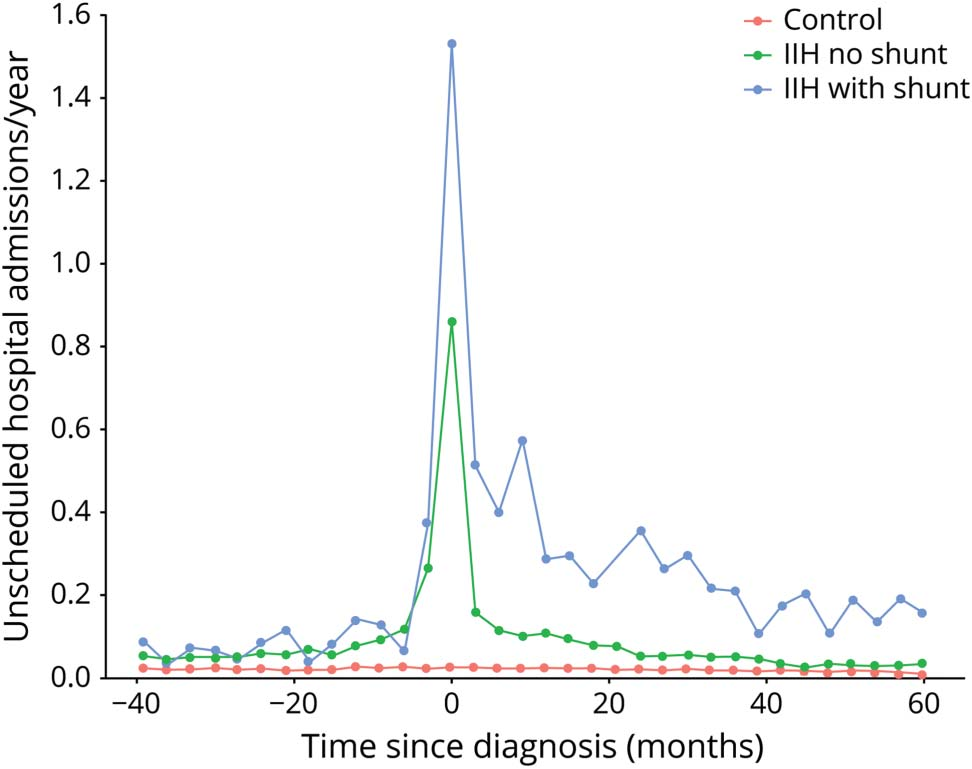
Rates of Unscheduled Hospital Visits Among 3 Cohorts Rates of unscheduled hospital visits (per person per year) versus time since idiopathic intracranial hypertension (IIH) diagnosis in months. A negative value indicates time before the diagnosis and a positive value indicates time after the diagnosis. Green line represents the IIH cohort; red line represents the control cohort (matched on age, sex, and deprivation quintile); blue line represents the IIH cohort with CSF diversion surgery (CSF shunts).

During the study period, the IIH cohort had 4,818 unscheduled hospital visits over 3,262,942 days (mean rate of 0.54/patient/year), considerably more than the 2,755 unscheduled hospital visits over 9,860,456 days in the control cohort (mean rate of 0.10/patient/year; rate ratio 5.28; 95% CI 5.04 to 5.54; *p* < 0.001). This amounts to 777 additional unscheduled hospital visits for the cohort of 1,765 patients with IIH when compared to controls (figure 5).

Patients with IIH who had CSF diversion surgery (n = 158) had 1,215 unscheduled hospital visits over 407,751 days (mean rate of 1.088 visits/patient/year). When compared to patients with IIH without CSF shunts, the rate ratio for unscheduled hospital visits was 2.02 (95% CI 1.89 to 2.15; *p* < 0.01) (figure 5).

## Discussion

We used a combination of primary and secondary care diagnosis codes in anonymized, routinely collected data to ascertain cases of IIH in this large, retrospective cohort study. Uniquely, we were able to validate our IIH case ascertainment method and found a sensitivity of 92% when identifying a linked set of confirmed IIH cases. Diagnostic codes used in the United Kingdom are considered to be accurate,^11^ but specific validation of UK primary and secondary IIH diagnosis codes, which we have performed here, is rare. It would seem reasonable to use similar methods in future epidemiologic studies.

We found a significant increase in IIH incidence and prevalence in Wales. The prevalence of IIH in Wales increased sixfold from 12/100,000 in 2003 to 76/100,000 in 2017 and the incidence of IIH increased threefold from 2.3/100,000/y in 2003 to 7.8/100,000/y in 2017. There were corresponding increases in obesity rates, using primary care BMI measurements, with 29% of the population being obese in 2003 compared to 40% in 2017.

Until recently, there has been a paucity of large-scale epidemiologic studies of IIH. Older studies have found the incidence of IIH to be 1–2/100,000.^12–14^ A recent meta-analysis of 15 studies, from 10 different countries, included 889 patients with IIH and found a pooled incidence of 1.2/100,000/y.^15^ Larger, more recent studies have found higher, and increasing, incidence rates of IIH, in line with our findings. A study using routinely collected English secondary care data identified 23,182 new cases of IIH (82.4% female) with an incidence of 2.3/100,000/y in 2002 and 4.7/100,000/y in 2016.^1^ Another recent study used English primary care data to study cardiovascular risk in women with IIH and found that the incidence of IIH in women increased from 2.5 in 2005 to 9.3/100,000/y in 2017.^16^ A Minnesota study also demonstrated over a 24-year period that IIH incidence was increasing in a highly correlated fashion with obesity.^4^

The considerable increase in IIH incidence is multifactorial but likely predominately due to rising obesity rates. The worldwide prevalence of obesity nearly tripled between 1975 and 2016 and therefore these results also have global relevance.^17,18^ The increase in IIH incidence may also be attributable to increased IIH diagnosis rates due to raised awareness of the condition and greater use of digital funduscopy at routine optometry appointments. Misdiagnosis of IIH may also contribute to increasing IIH incidence; for example, there can be issues when interpreting fundoscopy in patients with headache.^19^

We found a strong association between increasing BMI, sex (female), and IIH. Around 85% of our IIH cohort was female, similar to other studies.^1,19^ We also found a significant association with increased deprivation and IIH, particularly in women. A total of 52.6% of our IIH cohort came from the 2 most deprived quintiles, a very similar finding to a recent English study that had 52.3% of its cases from the 2 most deprived quintiles.^1^

The association between IIH and deprivation can be explained by increasing obesity rates in more deprived areas. However, results from our logistic regression model suggest that even when adjusting for BMI, there was still an association between IIH and deprivation in women, but not men. This result was also seen in a recent study of English women with IIH.^5^ We found that women were 1.5 times more likely to develop IIH in the most deprived compared to the least deprived areas, even after adjusting for BMI. In women it therefore seems likely that factors that are associated with deprivation, apart from obesity alone, also contribute to the etiology of IIH. Central adiposity is prominent in IIH and it may be that the distribution of fat changes with deprivation.^20^ Factors associated with deprivation such as diet, pollution, smoking, and psychosocial stress may have a metabolic or endocrine effect by, for example, increasing circulating androgen levels, which are associated with IIH and altered CSF flow.^21^

We found that for men, although our numbers were smaller, IIH is associated with BMI only and not deprivation. There was a weaker association of BMI with IIH and a smaller increase in IIH incidence with time in men when compared with women. Men are 4 times less likely to develop IIH than women after adjusting for BMI and deprivation. This adds to the evidence that IIH in men has different characteristics than IIH in women.^1^

We have demonstrated that people with IIH have increased rates of unscheduled health care utilization compared with a matched control cohort. The rate ratio for unscheduled hospital admissions in the IIH cohort compared to the controls was 5.28 (95% CI 5.04 to 5.54). A considerable proportion of this excess in unscheduled hospital admissions occurs at the time of diagnosis and can be explained by the need for urgent investigation of papilledema with brain imaging and spinal fluid analysis. However, there is also a considerable excess in unscheduled hospital admissions up to 2 years after diagnosis.

It is likely that these admissions are for severe headache. In the past, emergency admissions may have led to therapeutic lumbar punctures, although recent UK consensus guidelines advise against therapeutic lumbar punctures in the majority of cases.^22^ There is therefore some scope to reduce emergency admissions through better management of headache, patient education, and rapid access to outpatient specialist advice. Interestingly, the rate of unscheduled admissions is higher in the IIH cohort in the 3 years leading up to diagnosis, suggesting an opportunity for earlier diagnosis and earlier intervention.

About 9% of people with IIH in Wales receive CSF diversion procedures a mean of 1.33 years after diagnosis. Individuals with IIH who have undergone CSF diversion procedures also have significantly increased unscheduled health care admission rates compared with individuals with IIH who have not undergone CSF diversion procedures (rate ratio for unscheduled admissions 2.02, 95% CI 1.89 to 2.15). They have around a 40% chance of having at least 1 CSF shunt revision procedure.

Forty percent of our IIH cohort had a second BMI recording after diagnosis. In these 691 individuals, their mean BMI increased by 0.48 kgm^−2^ (*p* = 0.02, 95% CI 0.07 to 0.89). This corresponds to an increase of 1.3 kg (using an average height of 1.62 meters for women in Wales). This reflects the difficulty in body weight reduction despite weight loss being the main management strategy for IIH.^2^ Despite this, there were very low rates of bariatric surgery in our cohort. Although 9% had shunts, less than 0.3% had bariatric surgery to tackle the underlying obesity. Moreover, the shunt population still had high rates of hospital attendance despite invasive treatment.

We analyzed a large number (32 million patient-years) of population-level data, integrating both primary and secondary care datasets and obtaining a large cohort of IIH cases and matched controls. Using routinely collected data reduces recruitment bias seen in other studies. We also include routinely collected BMI measurements for the majority of our IIH cohort in addition to deprivation measures in our regression model.

This was a retrospective study. Routinely collected health care data are not primarily collected for research purposes, can be incomplete, and may contain inaccurate diagnosis codes. At the time of analysis, within SAIL we had access to 100% of the Welsh population's secondary care diagnosis codes but only 80% of the population's primary care data. BMI is not routinely measured by GPs, raising the possibility of ascertainment bias as those who have BMI recorded are more likely to have ongoing weight issues. We made an attempt to adjust for this by also looking at national questionnaire data for BMI, but these self-reported data are subject to underreporting of obesity. We used an area-based measure of deprivation (WIMD), which, like other commonly used deprivation markers, does not take into account individual levels of deprivation. We did not specifically record prescriptions of drugs such as tetracyclines and retinoids that can cause secondary intracranial hypertension although this probably accounts for a small number of cases.^23^

IIH incidence and prevalence in Wales are increasing considerably, corresponding to population increases in BMI. IIH is associated with increasing deprivation in women even after adjusting for obesity, suggesting additional etiologic factors associated with deprivation apart from BMI. This effect was not seen in men, pointing to sex-specific drivers for IIH.

The increasing incidence of IIH, together with the increased health care utilization in individuals with IIH and particularly those who have CSF shunts, have important implications for health care professionals and policy makers.

## Glossary

BMI: body mass index
CI: confidence interval
GP: general practitioner
ICD-10: International Classification of Diseases–10
IGRP: Information Governance Review Panel
IIH: idiopathic intracranial hypertension
LSOA: lower super output area
NSW: National Survey for Wales
SAIL: Secure Anonymised Information Linkage
WIMD: Welsh Index of Multiple Deprivation
